# Mapping cellular processes in the mesenchyme during palatal development in the absence of Tbx1 reveals complex proliferation changes and perturbed cell packing and polarity

**DOI:** 10.1111/joa.12425

**Published:** 2015-12-22

**Authors:** Lara J. Brock, Andrew D. Economou, Martyn T. Cobourne, Jeremy B.A. Green

**Affiliations:** ^1^ Department of Craniofacial Development and Stem Cell BiologyKing's College LondonLondonUK

**Keywords:** cell orientation, cell packing, cell proliferation, cleft palate, mesenchyme, Tbx1

## Abstract

The 22q11 deletion syndromes represent a spectrum of overlapping conditions including cardiac defects and craniofacial malformations. Amongst the craniofacial anomalies that are seen, cleft of the secondary palate is a common feature. Haploinsufficiency of TBX1 is believed to be a major contributor toward many of the developmental structural anomalies that occur in these syndromes, and targeted deletion of *Tbx1* in the mouse reproduces many of these malformations, including cleft palate. However, the cellular basis of this defect is only poorly understood. Here, palatal development in the absence of *Tbx1* has been analysed, focusing on cellular properties within the whole mesenchymal volume of the palatal shelves. Novel image analyses and data presentation tools were applied to quantify cell proliferation rates, including regions of elevated as well as reduced proliferation, and cell packing in the mesenchyme. Also, cell orientations (nucleus–Golgi axis) were mapped as a potential marker of directional cell movement. Proliferation differed only subtly between wild‐type and mutant until embryonic day (E)15.5 when proliferation in the mutant was significantly lower. *Tbx1* ^*−/−*^ palatal shelves had slightly different cell packing than wild‐type, somewhat lower before elevation and higher at E15.5 when the wild‐type palate has elevated and fused. Cell orientation is biased towards the shelf distal edge in the mid‐palate of wild‐type embryos but is essentially random in the *Tbx1* ^*−/−*^ mutant shelves, suggesting that polarised processes such as directed cell rearrangement might be causal for the cleft phenotype. The implications of these findings in the context of further understanding Tbx1 function during palatogenesis and of these methods for the more general analysis of genotype–phenotype functional relationships are discussed.

## Introduction

The deletion of chromosome 22q11 is the most common microdeletion seen in human populations, occurring with a frequency of about 1 : 4000 live births (McDonald‐McGinn et al. 1997; Botto et al. 2003) and associated with three distinct but overlapping conditions, all dominated by the presence of cardiac defects and craniofacial anomalies. The 22q11 deletion syndromes (22q11DS) encompass at least three clinical entities, including DiGeorge (DGS), velocardiofacial (VCFS) and conotruncal anomaly face (or Takao) syndromes (Scambler et al. 1991, 1992; Driscoll et al. 1992; Burn et al. 1993). Haploinsufficiency of *TBX1* is believed to be a major contributor toward many of the developmental structural anomalies that occur in 22q11DS. This candidature is based primarily upon chromosomal location (Chieffo et al. 1997; Jerome & Papaioannou, 2001; Lindsay et al. 2001; Merscher et al. 2001), embryonic expression pattern (Chapman et al. 1996; Zoupa et al. 2006) and findings that targeted deletion of *Tbx1* in the mouse can reproduce many of the malformations seen in 22q11DS (Jerome & Papaioannou, 2001; Lindsay et al. 2001; Merscher et al. 2001). Moreover, reduced *TBX1* function in humans has been associated with some forms of non‐deletion DGS/VCFS (Yagi et al. 2003; Stoller & Epstein, 2005; Paylor et al. 2006). An absence of *Tbx1* function in the mouse results in a severe DGS/VCFS phenotype, which includes loss of the third, fourth and sixth pharyngeal arch arteries, cardiac outflow tract disruption, thymus and parathyroid aplasia and cleft palate (CP; Jerome & Papaioannou, 2001; Lindsay et al. 2001; Merscher et al. 2001). More recently, varying combinations of inducible and tissue‐specific conditional mouse mutants have demonstrated a requirement for *Tbx1* during development of the pharyngeal region that is sensitive to dosage, timing and tissue‐type (Baldini, 2006; Scambler, 2010).

Amongst the craniofacial anomalies that can occur within the 22q11DS spectrum, oro‐facial clefting, micrognathia and ear abnormalities predominate. These are found in association with varying degrees of facial dysmorphology, which can be subtle but often include hypertelorism, up‐slanted and hooded eyes, long face, malar flatness and a blunted, full nose (Arvystas & Shprintzen, 1984; Lipson et al. 1991; Hammond et al. 2005). CP is seen in about 10% of subjects, making 22q11DS one of the most common causes of human syndromic CP (Ryan et al. 1997). In addition, both frank and occult submucous clefting, palatal and pharyngeal asymmetry and velopharyngeal insufficiency can occur, which may all contribute towards impaired palatal function in affected individuals (Shprintzen, 1982). In both humans and mice, the secondary palate forms from the paired palatine shelves, which descend vertically adjacent to the developing tongue as they grow from the maxilla. Subsequent shelf elevation takes them above the tongue, with further medial growth producing first contact and then fusion with their counterpart at the midline. The shelves also fuse with the nasal septum superiorly and the primary palate anteriorly, sealing and separating the oral and nasal cavities. In the mouse, this process occurs over a relatively short period of time and involves complex signalling interactions between epithelium and mesenchyme of the palatal shelves (Gritli‐Linde, 2008).

Mice lacking the function of *Tbx1* have CP with complete penetrance (Jerome & Papaioannou, 2001; Lindsay et al. 2001; Merscher et al. 2001), and current evidence suggests that both extrinsic and intrinsic factors are likely to be important etiological factors. These mice have mandibular retrognathia, a high tongue position and hypoplasia of the branchiomeric muscles (Kelly et al. 2004; Liao et al. 2004; Kong et al. 2014), whilst the palate epithelium is hyperproliferative and associated with abnormal adhesions to the oral surface of the mandible (Goudy et al. 2010; Funato et al. 2012). Collectively, these factors are likely to impede normal elevation of the palatal shelves. However, *Tbx1* is expressed within the palatal shelf epithelium throughout palatogenesis and the shelves are reduced in size in the mutant, failing to elongate appropriately, even in roller culture when the mandibular structures have been removed (Goudy et al. 2010; Funato et al. 2012). This is suggestive of an intrinsic growth defect within the palatal shelf mesenchyme, mediated through Tbx1 in the epithelium. To date, only proliferative activity and programmed cell death have been assayed in the secondary palate of *Tbx1* mutant mice and, whilst the numbers of TUNEL‐positive cells are approximately double in the mutant, the findings relating to proliferation are ambiguous, with reports of both significantly increased and reduced levels in the mesenchyme (Goudy et al. 2010; Funato et al. 2012).

Here, a semi‐automated method of image analysis was applied to measure cell behaviour in the entire *Tbx1* ^*−/−*^ palatal mesenchyme before, during and after shelf elevation; both to resolve the contradictions in reported proliferation abnormalities and to identify where non‐proliferation‐related defects may be important. This study focused on mesenchyme rather than epithelium because mechanically it is, by cellular mass, quantitatively predominant in the physical growth and elevation of the palate relative to epithelium. It was shown that cell packing is slightly lower in mutant than wild‐type tissue and that proliferation differences are subtle before embryonic day (E)15.5, with transiently higher levels in mutant at E11.5 and in wild‐type at E13.5. It was further shown that cell polarity, as marked by orientation of the Golgi apparatus (an anterior marker in migrating cells), is perturbed in *Tbx1* ^*−/−*^ palate mesenchyme, particularly in the posterior region, where cell rearrangement rather than a ‘flip up’ is thought to drive the elevation process (Ferguson, 1988; Bush & Jiang, 2012). Implications for the role of non‐proliferative process defects in the *Tbx1* ^*−/−*^ palate and more generally in this mutant phenotype are discussed.

## Materials and methods

### Mouse strains

All mice were housed in the Biological Services Unit at King's College London, and experiments conducted in compliance with the approved institutional and UK government protocols. *Tbx1* ^*−/−*^ mutant mice were generated and maintained in a C57BL6 background and genotyped as previously described (Lindsay et al. 2001). Timed‐matings were set up such that noon of the day on which vaginal plugs were detected was considered as E0.5.

### Proliferation assay

For proliferation assays, mouse embryos were labelled with iododeoxyuridine (IddU) or bromodeoxyuridine (BrdU) via intraperitoneal injection into pregnant females at 2.5 h prior to death (50 mg or 20 mg/100 g body weight, respectively). Injection with either label gave essentially identical results, and therefore only IddU data are shown here. Time‐delayed injection (Boehm et al. 2010) gave unexplained anomalously long cell cycle time calculations and so was not used.

### Fixing, sectioning and staining

Tissues were fixed in 4% paraformaldehyde overnight at 4 °C. For the IddU and cell spacing analyses, specimens were embedded in wax by standard methods, sectioned at 8 μm thickness and immunostained according to the manufacturer's instructions with anti‐IddU antibody (BD Biosciences) diluted 1 : 200. For Golgi orientation analyses, specimens were embedded in sucrose–gelatin and thick‐sectioned at 30 μm and immunostained according to the manufacturer's instructions with rabbit‐anti‐giantin antibody (Abcam) at a dilution of 1 : 500. Sections were stained with DAPI nuclear stain before mounting in Mowiol.

### Image acquisition

Images (1024 × 1024 pixels) were taken on a TCS SP5 Leica confocal microscope with a HCX PL APO CS N.A.1.25 × 40 oil immersion objective using Leica LAS‐AF software. For large tissue areas, multiple images were taken using a motorised XY stage and then stitched together using the LAS‐AF software. For cell spacing, single confocal planes were analysed. For nucleus–Golgi angles, image stacks were taken with confocal sections at 0.17 μm intervals.

### Location of nuclei and proliferation labelling by image segmentation

Images were imported into Volocity (Perkin‐Elmer) software and speckles removed using the Fine Filter. Nuclei were located by: (i) applying a global intensity threshold (manually adjusted to pick out nuclei but consistently at an intensity value of about 20); (ii) eroding the identified objects by 1 pixel to improve object separation; (iii) applying the Separate Touching Objects tool in Volocity; and (iv) excluding objects < 9 μm^2^ to remove the remaining noise. This method was validated manually against typical regions within 10 different sections, each containing 100–200 cells, and found to give accurate cell counts to within ± 4%. Volocity was used to output the centroid coordinates of nuclei segmented in this way for further analysis and mapping. IddU‐positive cells were scored by: (i) using the compartmentalise tool in Volocity to analyse only previously identified nuclei; (ii) applying a pixel intensity threshold of 10 but with the Volocity ‘local contrast adjustment’ option; (iii) applying a 9‐μm^2^ size filter to remove speckles and noise; (iv) applying ‘fill holes in objects’ followed by an object dilation and clipping to within the identified nuclei to consolidate the patchy IddU staining within nuclei; (v) applying a final 9‐μm^2^ size filter to remove speckles and noise. This protocol was validated against a panel of 10 typical images and found to match manual IddU counting ± 6% (data not shown). Centroid coordinates were output by Volocity for further analysis.

### Generation of cell packing and proliferation maps

Centroid coordinate data were entered into the statistical package R (R Development Core Team, 2011). For cell packing maps, macros written in R by the authors exploited the built‐in Delaunay triangulation algorithm to identify and draw line segments to all the nearest neighbours of each centroid. To provide some smoothing in the plane of the section, the length of the line segments from each centroid was averaged together with those from each of the nearest neighbours to their neighbours. This average was used as the measure of local cell packing. Images of three sections each 16 μm apart were averaged by first manually aligning the section images (using only translation and rotation) to determine the necessary transformations, applying the respective transformations to the centroid datasets, concatenating the datasets, re‐triangulating nearest neighbours and re‐averaging. Output functions in R were used to apply a colour scale from the generated spacing values to Voronoi tiles around each centroid.

For cell proliferation maps, R was used to calculate a local labelling index for a region around each cell (centroid). This region was defined as the cell plus its nearest neighbours plus their nearest neighbours (i.e. the cell plus a double corona of surrounding cells) and the index was the fraction of label‐positive cells in that region. Three adjacent sections were then averaged and colours assigned to Voronoi tiles to generate heat maps, as above.

Regional maps and histogram data were obtained by manually cropping the relevant regions and averaging the data values within them. The macro code used for the above R procedures is available on request.

### Generation of nucleus–Golgi orientation data

Nuclei and Golgi apparatuses were located in image stacks manually. Line segments were drawn between their centres using Volocity and the projection of the lines onto the frontal plane recorded. Angle data were binned into 30 ° bins and the square root of frequencies plotted using an adaptation of the Radar Plot in Microsoft Excel, so that the frequency corresponds to the area of the plotted sectors rather than their radial height (which would exaggerate small differences).

### Statistical analysis

The mean angle and statistical significance of the results were calculated in R using the free package ‘CircStats’ (Agostinelli, 2009). The non‐parametric Watson's U^2^ test was used to test the dataset against a circular uniform distribution (Mardia, 1975; Bergin, 1991; Concha & Adams, 1998), and Watson's two‐sample test of homogeneity was used to test for significant differences between different datasets. The multiple measures (anova equivalent) is not well developed for circular statistics, and so the *P*‐values presented must be treated very conservatively to reduce the risk of inferring false significance by chance.

## Results

### Palatal shelf dimensions in *Tbx1* ^*−/−*^ mice are different from but not always smaller than those in wild‐type

**Figure 1:**
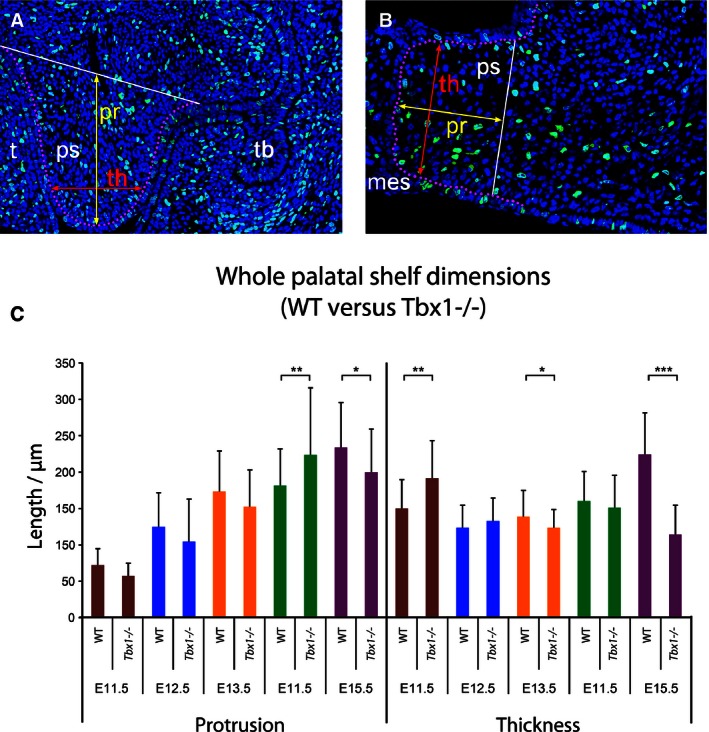
Gross palate dimensions in WT and *Tbx1* ^*−/−*^ embryos. (A, B) Representative frontal sections of the developing palate at E13.5 and E14.5, respectively, marked to show landmarks for gross measurements. The palatal shelf boundary was constructed between the two ‘axils’ of the palatal outgrowth prior to elevation at E13.5 or between the nasal shoulder and the nearest oral epithelial surface at E14.5 (white line in A and B, respectively). Protrusion of the palatal shelf was taken as the length of a line constructed from the distal edge of the shelf through the centre and perpendicular to the shelf boundary (yellow arrowed line in A and B, respectively). The thickness of the palatal shelf was measured as the length of a line constructed at right‐angles to the protrusion reference line one‐quarter of the total protrusion length away from the distal edge. The mesenchymal compartment is outlined with purple dots. mes, medial epithelial seam; ps, palatal shelf; pr, protrusion; t, tongue; tb, tooth bud; th, thickness. (C) Palate protrusion and thickness in WT and *Tbx1* ^*−/−*^ mutant mice. Mutant palatal shelves are initially thicker (E11.5) and later transiently protrude more (at E14.5) than in WT. Significant differences indicated are *P < 0.05, **P < 0.01, ***P < 0.001 by *t*‐test.

In order to investigate the basis of CP in the absence of *Tbx1* function, first the gross tissue dimensions of palatal shelves derived from wild‐type and *Tbx1* ^*−/−*^ mice at E11.5–15.5 were compared, estimating both shelf protrusion from the maxilla and shelf thickness during outgrowth, elevation and approximation (Fig. 1). When measurements from intermittent serial frontal sections were averaged over the whole antero‐posterior (AP) extent of the palate there was, as one would expect, a trend for palatal shelf protrusion to increase with development in both wild‐type and mutant. Between E11.5 and E13.5, the mutant palate protruded less than the wild‐type, although this was not statistically significant. Interestingly, at E14.5 the mutant palate protruded significantly more than the wild‐type, perhaps because wild‐type shelves are in the process of elevating at this stage, but by E15.5 this difference had been reversed and the mutant shelf protruded significantly less. In relation to shelf thickness, there was initially some thinning of the wild‐type shelves between E11.5 and E12.5, followed by a general thickening thereafter. The mutant shelves were thicker than wild‐type during early palatogenesis (E11.5–E12.5); however, from E13.5 to E15.5 the shelves were thinner in the mutant, which was significant at both E13.5 and E15.5. Breaking down the measurements of protrusion and thickness into three regions along the AP axis of wild‐type and mutant palatal shelves revealed similar trends throughout (data not shown). Collectively, these data demonstrate significant differences in palatal shelf dimensions between wild‐type and *Tbx1* ^*−/−*^ mice during the process of palatogenesis, particularly between E13.5 and E14.5 during palatal shelf elevation. Strikingly, although the mutant palatal shelves were smaller than in wild‐type by E15.5, this was not the case throughout palate development and, in fact, the mutant shelves were significantly longer than wild‐type at E14.5.

### Cell packing in wild‐type and *Tbx1* ^*−/−*^ mice

Having established the presence of gross dimensional variations in the secondary palate of developing *Tbx1* ^*−/−*^ mice, it was sought to investigate these changes in relation to the cellular properties of this tissue. To this end, next heat maps were generated for internuclear distances to measure local cell packing changes (capturing both cell size change and changes in extracellular matrix) in the mutant. The analysis was carried out in five steps. First, fluorescence images of the sections in the blue (DAPI‐stained) channel were segmented to identify nuclei as individual objects. Using the segmented images, the centroids of these nuclei were identified and used as the nodes in a Delaunay triangulation. Then, the average length of the triangulation lines emerging from each node was calculated. Finally, colours were assigned to the values of those averages and those colours were applied to a Voronoi‐tiled map of the original image, in which each tile corresponds to the location of each centroid.

**Figure 2:**
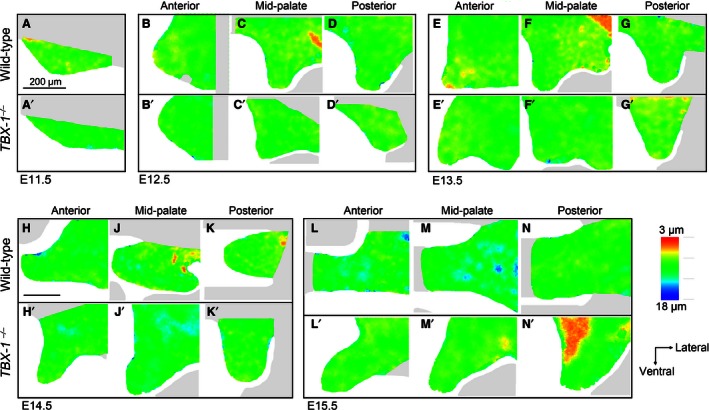
Cell packing heat maps of E11.5–E15.5 wild‐type and *Tbx1* ^*−/−*^ palatal shelf mesenchyme reveals AP differences in cell spacing. Cell packing heat maps at (A–A′) E11.5; (B–D′) E12.5; (E–G′) E13.5; (H–K′) E14.5; (L–N′) E15.5. Each heat map represents an average of three individual sections and is representative of at least three different embryos at each stage. Scale bar: 200 μm (A, and applies to all panels).

At E11.5, the mutant palate was slightly less densely packed than the wild‐type, consistent with the increased width (thickness) seen at this stage (Fig. 2A′ vs. 2A, noting less yellow and a slightly darker shade of green). Cell packing increased in the wild‐type from E11.5 to E13.5, particularly in the anterior and middle regions (Fig. 2B–D, E–G). In contrast, the *Tbx1* ^*−/−*^ mesenchyme showed no such increase in the anterior and middle regions, but instead a slight increase in posterior cell packing (Fig. 2B′–D′, E′–G′). At E14.5, in both the elevated wild‐type and non‐elevated mutant shelves, the cell density was decreased, particularly in the core of the middle palate, with no clear differences in the distribution of cell packing between genotypes. At E15.5, where the shelves had fused in the wild‐type, there were further reductions in density, mainly within the anterior and middle regions, which was not seen in the mutant.

Mapping revealed variation in local cell packing to be, in general, quite noisy. It can vary from about 7 to 14 μm (yellow to cyan in Fig. 2) between stages, although the variation at a given stage is probably about half this range. Overall, the cell packing data revealed a complex difference between wild‐type and mutant palate mesenchyme: the AP organisation of the tissue was subtly disrupted, the progression of increasing density before elevation is retarded and the decreasing density after elevation in the wild‐type is absent.

### Regionalised proliferation during palatogenesis in wild‐type and *Tbx1* ^*−/−*^ mice

In order to further understand the influence of Tbx1 function during growth and development of the secondary palate, regionalised heat maps of cellular proliferation were constructed from E11.5–E15.5 in wild‐type and *Tbx1* ^*−/−*^ mutant palatal shelves. This was done using IddU labelling and calculating a local labelling index for each nucleus as the proportion of labelled nuclei in an area consisting of that nucleus plus two concentric rings of its nearest neighbouring nuclei.

**Figure 3:**
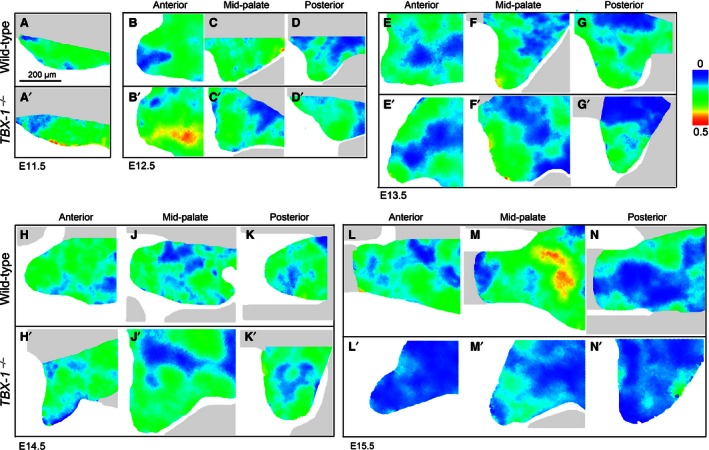
Proliferation heat maps of E11.5–E15.5 wild‐type and *Tbx1* ^*−/−*^ palatal shelf mesenchyme reveals severely reduced proliferation at E15.5 superimposed on high local variability. Proliferation heat maps at (A–A′) E11.5; (B–D′) E12.5; (E–G′) E13.5; (H–K′) E14.5; (L–N′) E15.5. Each heat map represents an average of three individual sections and is representative of at least three embryos at each stage. Scale bar: 200 μm (A, and applies to all panels).

Unlike the previous analysis of proliferation in the secondary palate epithelium (Economou et al. 2013), the proliferation index heat maps of palatal shelf mesenchyme showed a remarkably noisy and inconsistent pattern at all stages for both mutant and wild‐type. Figure 3 shows illustrative examples for each stage, genotype and AP region. This suggests that the cell cycle *in vivo* may be unexpectedly stochastic, smoothing out only over longer periods than were used for labelling, or possibly being asynchronous in a way that rendered IddU labelling more variable than in other tissues. However, against this high local variability, the maps did show a dramatic difference in proliferation between *Tbx1* ^*−/−*^ mutant and wild‐type at E15.5 (Fig. 3L′–N′ vs. L–N). This unmistakable large‐scale difference was relatively late compared with other aspects of the mutant phenotype.

**Figure 4:**
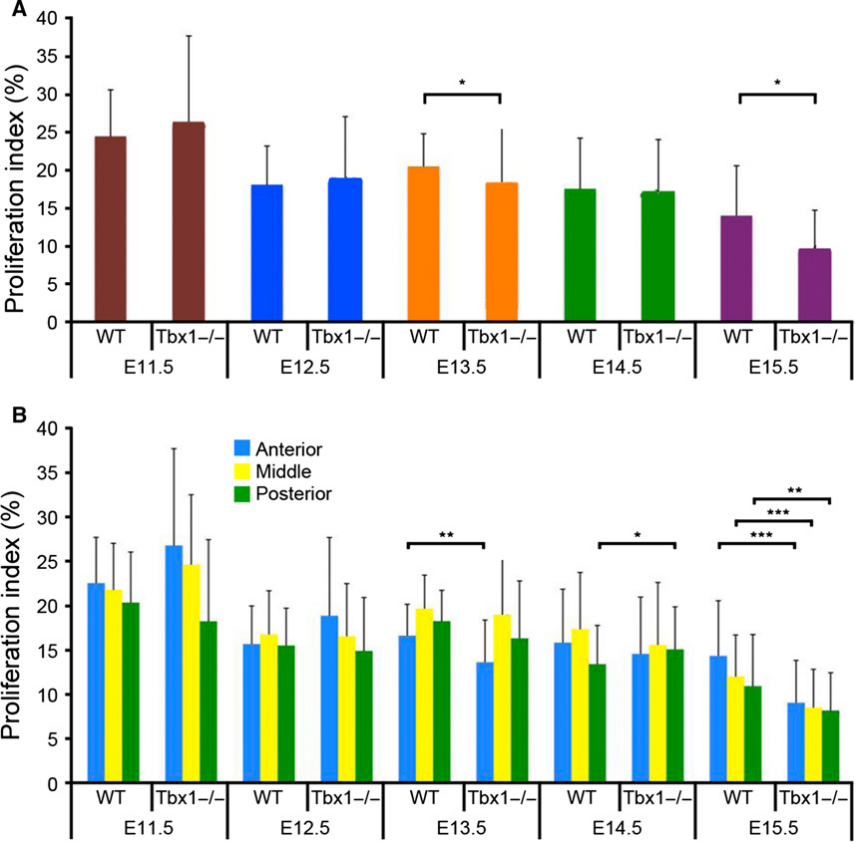
Histograms of proliferation indices for palate mesenchyme in E11.5–E15.5 wild‐type and *Tbx1* ^*−/−*^ mutants. (A) Proliferation indices averaged over the whole mesenchyme, and (B) the anterior, middle and posterior thirds of the mesenchyme. Error bars are ± SD. Significant differences indicated are *P < 0.05, **P < 0.01, ***P < 0.001 by *t*‐test.

Given the local noisiness of the maps, it was chosen to quantify the regional proliferation rates over larger regions of the mesenchymal tissue. Averaging the proliferation index at each stage between E11.5 and E15.5 demonstrated a general trend in the wild‐type towards reduced levels of proliferation as palatal development proceeded, albeit with a slight increase at E13.5, which was not observed in the *Tbx1* ^*−/−*^ mutant (Fig. 4A). Interestingly, proliferation was higher in the mutant than in wild‐type at E11.5, although this was not statistically significant (*P *> 0.05, *t*‐test). Whilst there was no significant difference in proliferation between wild‐type and mutant at E14.5, there was at E15.5, consistent with the visual impression from the heat maps. Proliferation was further investigated considering anterior, middle and posterior regions of the palate mesenchyme separately. Not surprisingly, the proliferation index values in the three regions broadly reflected the changes seen in the overall index, namely a general trend towards reduced levels over time, but with an increase in the wild‐type from E12.5 to E13.5 that was not seen in the mutant. However, the reduced proliferation seen in the mutant compared with wild‐type in all regions at E15.5 was more statistically significant (Fig. 4B). This effect is indicative of a high degree of noise in the data, which may reflect a genuine variability in proliferation rates *in vivo* in this tissue, but underlines the surprisingly late emergence of a proliferation deficit in the *Tbx1* ^*−/−*^ mutant.

### Cell polarity in wild‐type and *Tbx1* ^*−/−*^ mice

Growth and morphogenesis can depend not only on cell proliferation and density, but also on non‐proliferation‐related processes, such as cell rearrangement. In order to identify potential non‐proliferation‐related defects in cell behaviour as a contributor to the CP phenotype in *Tbx1* ^*−/−*^ mice, Golgi orientation within the palatal shelves was also investigated. The Golgi apparatus is an anterior marker in migrating cells, and therefore serves as a proxy marker for potential cellular migration.

**Figure 5:**
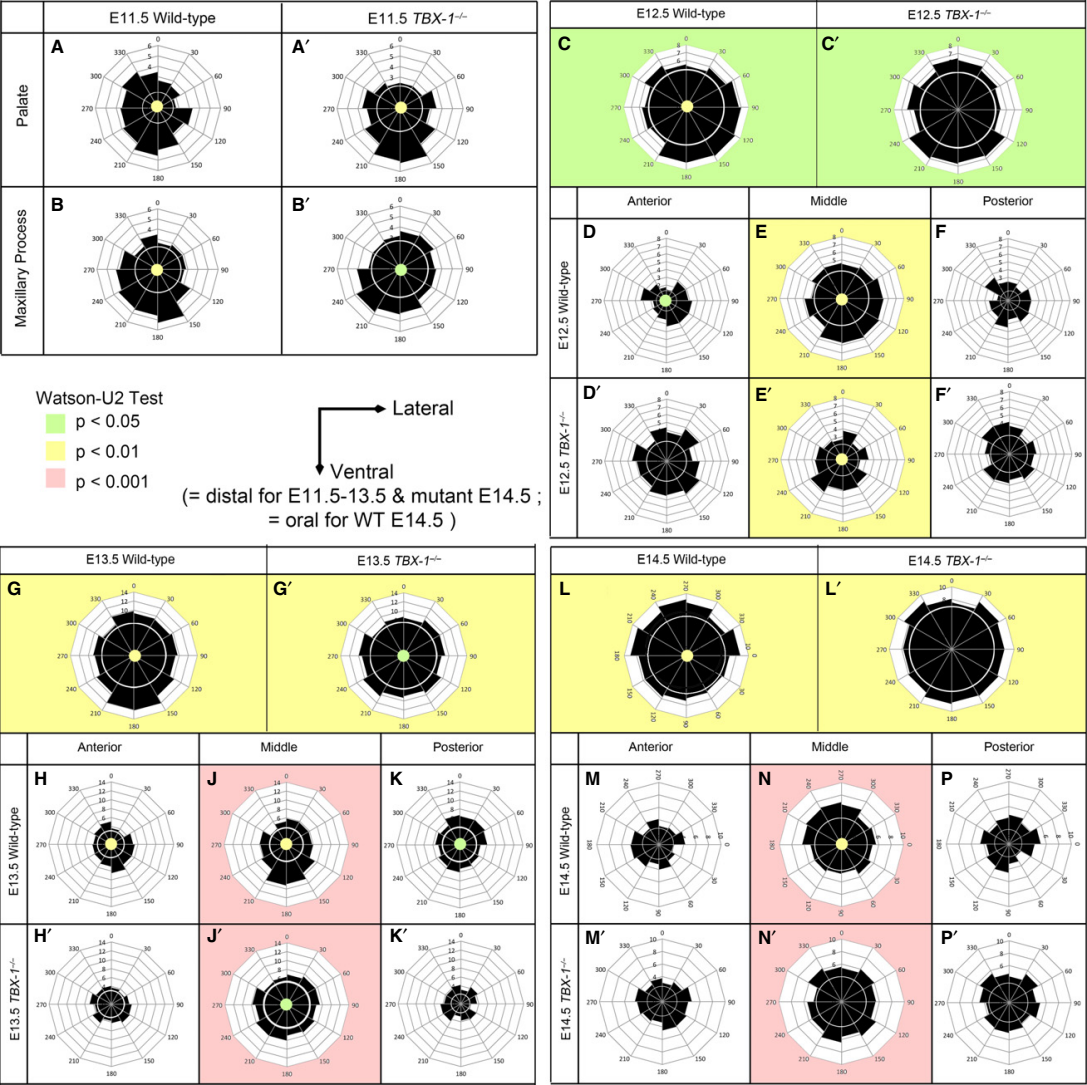
Cell orientation distributions show aberrant and weaker directional biases in *Tbx1* ^*−/−*^ mutant than in wild‐type palate mesenchyme. Rosette diagrams (circular histograms) depict the nucleus–Golgi angles (scored in 30 ° bins) relative to body axes in whole palatal shelves (A, A′, C, C′, G, G′, L,L′), adjacent maxilla (B, B′), or anterior, middle and posterior thirds of the palatal shelf mesenchyme (D–F′, H–K′, M–P′) for wild‐type (A–P) and *Tbx1* ^*−/−*^ mutant (A′–P′) embryos, respectively. Each panel represents data from at least three embryos. Light‐coloured circles at the centre of a rosette indicate statistical significance from a uniform distribution (*P*‐values indicated by colour, as shown). Coloured backgrounds in adjacent panels indicate statistically significant differences between them at the *P*‐values indicated. White rings are a visual aid to show deviations from a uniform distribution. For statistical tests, angles were taken relative to the distal edge, i.e. ventral in all cases except the elevated E14.5 wild‐type where the distal edge is medial (note the rotated angle scale on these plots, with ‘180 °’ to the left rather than down).

During initial palatal outgrowth at E11.5, both wild‐type and mutant shelves demonstrated non‐uniform palatal distributions, with an evident ventral and medial bias in both palatal outgrowth and the adjacent maxillary mesenchyme (Fig. 5A,B′). At this stage, mutant and wild‐type were not statistically significant. At E12.5, the overall distribution of Golgi in the wild‐type was directed ventro‐laterally, whilst in the mutant the distribution was statistically different and indistinguishable from random (although with a weak modal ventral orientation; Fig. 5C vs. C′). Considering AP sub‐regions of the palate, the middle thirds from both mutant and wild‐type were significantly different from uniform, but also significantly different from each other: mutant orientation was predominantly ventro‐medial whilst in wild‐type it was ventro‐lateral (Fig. 5E vs. E′).

At E13.5, coherent non‐uniform orientation in the wild‐type became more pronounced and significant (Fig. 5G). Orientation in the *Tbx1* ^*−/−*^ mutant was significantly different from wild‐type (Fig. 5G vs. 5G′). However, in the wild‐type posterior palate at E13.5, the cells appeared to have a roughly dorsal distribution (i.e. an almost 180 ° reversal of orientation), which differed significantly from the predominantly ventral bias of the middle and anterior palate at that stage, consistent with the notion that the posterior palate is about to undergo elevation via dorsal cell movement.

At E14.5, following palate elevation in the wild‐type, the predominant Golgi orientation had moved somewhat more than 90 ° (from ventral to dorso‐medial, respectively) consistent with a whole‐tissue rotation or ′flip‐up’ mechanism (Fig. 5L vs. G). This was particularly apparent in the middle region, where the orientation was most biased to start with (Fig. 5J,N). Posteriorly, cell orientations were spread out compared with E13.5 to include medial as well as dorsal, consistent with an ongoing tissue remodelling process extending the palate medially by cell rearrangement. In the mutant, by contrast, no elevation or rotation had occurred, and Golgi orientations were statistically random (Watson U‐squared test vs. uniform distribution). The difference between mutant and wild‐type was significant in the middle region of the palate, even when comparing angles relative to the distal edge of the shelves (medial in wild‐type but still ventral in *Tbx1* ^*−/−*^; Fig. 5N,N′ – note rotated angle scale).

Finer‐grained mapping of Golgi orientation did not reveal any additional trends (data not shown).

## Discussion

The analyses detailed above begin to reveal the fine structure of the *Tbx1* ^*−/−*^ CP phenotype compared with the normal palatogenesis seen in wild‐type siblings. They raise two significant points. First, this phenotype is surprisingly complex and may be much more to do with cell orientation and cell packing defects than changes in cell proliferation. This complexity has previously been hinted at indirectly by the discrepant literature relating to variations in proliferation between *Tbx1* ^*−/−*^ and wild‐type palatal shelves (Goudy et al. 2010; Funato et al. 2012). Indeed, this study shows that the mutant has both increased and decreased proliferation, depending on the time and anatomical location of the analysis. This is hard to interpret, but is unambiguously an argument for full proliferation mapping in general, rather than the traditional method of sampling a few arbitrary squares of tissue to generate a proliferation index, commonly using BrdU. It can now be more confidently concluded that the smaller palate size is at least in part caused by a predominant reduction in proliferation.

The current data also identify novel aspects of the *Tbx1* ^*−/−*^ CP phenotype, specifically slight changes in cell packing and more obvious and dramatic changes in cell (Golgi) orientation. The changes in cell packing are currently hard to interpret, but the changes in orientation are rather simple: the normally coherent ventral (hinge‐to‐edge) orientation is randomised and the intriguing re‐orientation in the posterior palate concomitant with the process of elevation is effectively absent. It is known that the *Wnt5A* ^*−/−*^ mutant has a CP phenotype in which directional cell migration is disrupted (He et al. 2008). It will be interesting to investigate the tissue properties described here in *Wnt5A* ^*−*/*−*^ palate mesenchyme. Complementarily, it now makes sense to determine whether targets of Tbx1 include polarity genes in palate mesenchyme. Intriguingly, Tbx1 has recently been implicated in cell polarity defects in heart epithelium (Francou et al. 2014), suggesting that polarity regulation may be an important mechanism of action of the Tbx1 protein.

The second point raised by this study is that this level of analysis is possible and will be essential to provide the explanatory mechanistic link between genes and tissues. Many of the most dramatic mutant phenotypes, and certainly most birth defects, are noticed as defects in tissue architecture. This means that they are failures of growth and movement rather than of differentiation, as such. Growth itself is typically not isotropic expansion by proliferation, but involves directional increase. It has previously been shown in palate epithelium that a significant proportion of the AP growth in that tissue is not proliferation‐based, but rather arises by cell shape change and cell rearrangement (Economou et al. 2013). In the present paper, a preliminary study has been described, as methods for capturing the separate components of directional growth in three dimensions and in mesenchyme are still in development. Nonetheless, this first foray into tissue properties has already implicated cell orientation and, to a lesser extent, cell packing as major contributors to the *Tbx1* ^*−/−*^ mutant CP phenotype. It would be informative to apply these same methods to analysis of other anatomical loci of the phenotype, for example the heart. The current analysis provides an analytical, albeit still partial, picture of dynamic mechanisms that generate tissues. The missing factor is the observation and measurement of cell movement and rearrangement. Live imaging in deep tissue is non‐trivial, although increasingly feasible with multiphoton optics. However, in the case of the secondary palate, this is hampered by the lack of normal growth observed in the secondary palate cultured *in vitro* (Economou et al. 2012). Culture conditions with tractable optics remain an important objective. Meanwhile, clonal lineage tracing provides an excellent potential alternative that can be used in fixed material (Buckingham & Meilhac, 2011). Analytical tools to translate experimentally generated clonal patterns into growth mapping are, however, not yet well established.

## Conclusions

There were significant differences in palatal shelf dimensions between wild‐type and mutant, particularly during shelf elevation. Although the mutant shelves were smaller than wild‐type by E15.5, this was not the case throughout palatogenesis. There was a subtle disruption of cell packing in the mutant compared with wild‐type, with retarded progression of the increasing cell density seen pre‐elevation and an absence of the decreasing cell density seen post‐elevation. The proliferation indices indicated a general trend in the wild‐type towards steadily declining levels during palatogenesis, albeit with an increase from E12.5 to E13.5, which was not observed in the mutant. Greater differences were seen at E15.5, too late to be causal for clefting. During initial palatal outgrowth there was a non‐uniform cell polarity in both wild‐type and mutant shelves; however, prior to shelf elevation the non‐uniform orientation became pronounced in the wild‐type, which was not seen in the mutant. At this stage in the wild‐type, cells in the posterior palate had a roughly dorsal distribution, which differed significantly from the predominantly ventral bias of the middle and anterior palate. Following palate elevation in the wild‐type, the predominant cell orientation moved from ventral to dorso‐medial, consistent with a whole‐tissue rotation. In contrast, no elevation or rotation occurred in the mutant and cell polarity remained statistically random.

## Author contributions

LJB and ADE conducted the experiments, and carried out the computational analyses. JBAG and MTC designed the experiments. LJB, MTC and JBAG wrote the manuscript.
